# Up-Regulation of TRIM32 Associated With the Poor Prognosis of Acute Myeloid Leukemia by Integrated Bioinformatics Analysis With External Validation

**DOI:** 10.3389/fonc.2022.848395

**Published:** 2022-06-08

**Authors:** Xiaoyan Xu, Jiaqian Qi, Jingyi Yang, Tingting Pan, Haohao Han, Meng Yang, Yue Han

**Affiliations:** ^1^ National clinical research center for hematologic diseases, Jiangsu Institute of Hematology, The First Affiliated Hospital of Soochow University, Suzhou, China; ^2^ Institute of Blood and Marrow Transplantation, Collaborative Innovation Center of Hematology, Soochow University, Suzhou, China; ^3^ Department of Hematology, Key Laboratory of Thrombosis and Hemostasis of Ministry of Health, Suzhou, China; ^4^ State Key Laboratory of Radiation Medicine and Protection, Soochow University, Suzhou, China

**Keywords:** acute myeloid leukemia, weighted gene co-expression network analysis, differential gene expression analysis, TRIM32, prognosis

## Abstract

**Background:**

Acute myeloid leukemia (AML) is a malignant and molecularly heterogeneous disease. It is essential to clarify the molecular mechanisms of AML and develop targeted treatment strategies to improve patient prognosis.

**Methods:**

AML mRNA expression data and survival status were extracted from TCGA and GEO databases (GSE37642, GSE76009, GSE16432, GSE12417, GSE71014). Weighted gene co-expression network analysis (WGCNA) and differential gene expression analysis were performed. Functional enrichment analysis and protein-protein interaction (PPI) network were used to screen out hub genes. In addition, we validated the expression levels of hub genes as well as the prognostic value and externally validated TRIM32 with clinical data from our center. AML cell lines transfected with TRIM32 shRNA were also established to detect the proliferation *in vitro*.

**Results:**

A total of 2192 AML patients from TCGA and GEO datasets were included in this study and 20 differentially co-expressed genes were screened by WGCNA and differential gene expression analysis methods. These genes were mainly enriched in phospholipid metabolic processes (biological processes, BP), secretory granule membranes (cellular components, CC), and protein serine/threonine kinase activity (molecular functions, MF). In addition, the protein-protein interaction (PPI) network contains 15 nodes and 15 edges and 10 hub genes (TLE1, GLI2, HDAC9, MICALL2, DOCK1, PDPN, RAB27B, SIX3, TRIM32 and TBX1) were identified. The expression of 10 central genes, except TLE1, was associated with survival status in AML patients (*p*<0.05). High expression of TRIM32 was tightly associated with poor relapse-free survival (RFS) and overall survival (OS) in AML patients, which was verified in the bone marrow samples from our center. *In vitro*, knockdown of TRIM32 can inhibit the proliferation of AML cell lines.

**Conclusion:**

TRIM32 was associated with the progression and prognosis of AML patients and could be a potential therapeutic target and biomarker for AML in the future.

## Introduction

Acute myeloid leukemia (AML) is the most common type of acute leukemia in adults and is a heterogeneous disease both molecularly and clinically (1). It is a malignant disease of hematopoietic stem cells characterized by clonal proliferation of immature bone marrow cells blocked at different stages of differentiation (2). The accumulation of uncontrolled leukemic blasts often disrupts normal hematopoietic function, causing severe bleeding, infection, and anemia. Therefore, timely diagnosis and prompt treatment of AML are crucial. Currently, chemotherapy and allogeneic hematopoietic stem cell transplantation (allo-HSCT) are the main treatments for AML (3). However, the prognosis of AML patients is still unsatisfactory. Finding specific molecular targets for different patients is significant for the treatment outcome and long-term prognosis of AML.

TRIM32 (Tripartite motif-containing protein 32) is a member of the Tripartite motif protein family, first identified in 1995 as a protein binding to a key activator of viral transcription HIV-1 Tat (4, 5). It is a kind of ubiquitin ligase enzyme (E3) modulating the ubiquitination of proteins. TRIM32 is known to play a crucial role in skeletal muscle stem cell differentiation and to contribute significantly to muscle homeostasis (6, 7). TRIM32 is also involved in various biological processes such as cell growth, apoptosis and immunity (8). As tumor biology continues to advance, the relationship between TRIM32 and tumor progression is receiving increasing attention. The expression of TRIM32 is upregulated in several malignancies such as gastric cancer (GC), non-small-cell lung cancer (NSCLC), and hepatocellular carcinoma (HCC) (9–11). A recent study showed that elevated expression of TRIM32 in triple-negative breast cancer (TNBC) was associated with radiation resistance and poor prognosis (12). Targeted therapy against TRIM32 could increase radiosensitivity, which may be a new potential strategy. To date, the relationship between TRIM32 and AML has not been investigated.

With the rapid development of genomic and proteomic technologies, bioinformatics has facilitated the discovery of reliable biomarkers for disease diagnosis and prognosis. Correlation networks are increasingly used in bioinformatics applications. Weighted gene co-expression network analysis (WGCNA) is one of the most compelling analyses, which was first developed in 2008 (13, 14). Rather than focusing on a single gene or an isolated biomarker, WGCNA extracts gene co-expression modules and relates them to clinical features, increasing the sensitivity to identify potential worthwhile targets for biological regulation (15). Differential gene expression analysis has recently emerged as another effective tool to indicate key driver genes for diseases (16). Therefore, combining WGCNA with differential gene expression analysis can help identify potential biomarkers for diagnosing and treating specific diseases.

In this study, we extracted mRNA expression data of AML from TCGA and GEO databases and combined WGCNA and differential gene expression analysis to find differential co-expression genes of AML. More importantly, we used functional enrichment analysis and protein-protein interaction (PPI) network to screen out hub genes. We attempted to explore the potential role of TRIM32 in AML by integrated bioinformatics analysis and the results were validated by external data from our center. Then we knocked down TRIM32 in AML cell lines to verify its function, aiming to provide a potential biomarker for future diagnosis and treatment of AML.

## Materials and Methods

### Data Filtering and Processing

AML-related gene expression profiles and clinical data were extracted from the TCGA (https://portal.gdc.cancer.gov/) and GEO (https://www.ncbi.nlm.nih.gov/gds) databases. A total of 151 AML patients with RNA-seq data were enrolled from the TCGA database, which were downloaded by the *TCGAbiolinks* R package (17). As suggested by the tutorial, data filtering was performed using function *rpkm* in *edgeR* package (18).

After searching from the GEO database, another five datasets with survival outcomes were downloaded using R package *GEOquery* in this study (GSE37642, GSE76009, GSE16432, GSE12417, and GSE71014) (19). The number of patients in these five datasets was as follows: 562 in GSE37642, 534 in GSE76009, 436 in GSE16432, 405 in GSE12417, and 104 in GSE71014, respectively. As a result, a total of 2041 patients from the GEO database were included for subsequent analysis.

### Weighted Gene Co-Expression Network Analysis

To improve the accuracy of network construction, the genes used for WGCNA were filtered. The R package *WGCNA* was used to conduct WGCNA between the gene expression data profiles of TCGA-LAML and GEO datasets, grouping highly co-expressed genes into modules (14). We use *pickSoftThreshold* to establish a scale-free network. A similarity matrix was built after performing the Pearson correlation of all gene pairs. The adjacency matrix was then transformed into a topological overlap matrix (TOM) and the TOM-based dissimilarity matrix for hierarchical clustering. Then, we correlated previously computed module features with clinical characteristics to identify the co-expression network’s functional modules.

### Differential Gene Expression Analysis

To figure out the differentially expressed genes (DEGs) between AML patients with different survival status (alive or dead), the R package *limma* was applied in the TCGA and GEO databases. The DEGs were screened with the criteria |logFC| ≥ 1.0 and adj. P-value < 0.05. The R package *ggplot2* and *VennDiagram* were used to draw a volcano map and Venn diagram (20).

### Function and Pathway Enrichment Analysis

To determine the functional relevance of the modules, we tested whether the selected genes from the modules were enriched for specific functions or signaling pathways. Gene ontology (GO) and Kyoto Encyclopedia of Genes and Genomes (KEGG) pathway analyses were performed by the *clusterProfiler* R package (21). *p*<0.05 was considered statistically significant.

### Construction of PPI and Identification of Hub Genes

We used the online database *STRING* (https://string-db.org/) to construct a protein-protein interaction (PPI) network. The PPI network was analyzed and visualized using *Cytoscape* software (v3.7.2) with an extraction score ≥ 0.4. In the PPI network, the nodes stand for proteins, and the edges represent the interactions of proteins. To identify the hub genes from the PPI network, we used the maximal clique centrality (MCC) algorithm calculated by a plugin in *Cytoscape* named *CytoHubba.* Genes with top 10 MCC values were set as hub genes.

### Validation of the Expression Level and Prognostic Value of Hub Genes

To verify the reliability of the hub genes, we analyzed the expression level of hub genes in AML patients from the GEO database with different survival status (alive or dead). The expression level was represented with a boxplot for each gene. Univariate Kaplan-Meier survival analysis was done with R package *survival* to identify the prognostic value of hub genes further. Overall survival (OS) was defined as the time from diagnosis to death, regardless of the causes or last follow-up. Relapse-free survival (RFS) was defined as the time from complete remission to recurrence. Statistical significance was determined by Student’s t-test for two groups and one-way ANOVA was used for comparison among multiple groups. *p*<0.05 was regarded as statistically significant.

### Cells and Reagents

Human myeloid leukemia cell lines (MV4-11, MOLM13, KASUMI-1, OCI-AML3, SKM1, THP-1) were obtained from the American Tissue Culture Collection (ATCC). All cells were cultured in RPMI 1640 (Gibco, Detroit, MI, USA) medium, supplemented with 10% fetal bovine serum (Gibco, Detroit, MI, USA), 1% penicillin-streptomycin (Gibco, Detroit, MI, USA) at 37 ˚C with 5% CO_2_.

### Patient Samples and Characteristics

Bone marrow samples were obtained from 46 patients with newly diagnosed AML and 11 healthy donors (HD) from the First Affiliated Hospital of Soochow University between April 2020 and March 2021. The induction chemotherapy is a standard first-line treatment including an IA/DA regimen (Either idarubicin 8-12 mg/m^2^ or daunorubicin 60-90 mg/m^2^ on days 1-3 and cytarabine at 100mg/m^2^ on days 1-7). For some older patients or patients with organ dysfunction, CAG or revised CAG (IAG, HAG) were administered with or without hypomethylating agents. Bone marrow mononuclear cells were isolated by Ficoll density gradient centrifugation. Basic clinical information, including age, gender, white blood cell count, hemoglobin level, platelet count, bone marrow morphology, karyotype, and molecular information was acquired from each patient. Patient risk stratification was based on 2017 ELN genetic categories. Receiver operating characteristics (ROC) curve analysis was performed based on the patient survival status to determine the optimal cut-off value of TRIM32. Informed consent was available from all patients and approved by the Medical Ethics Committee of the First Affiliated Hospital of Soochow University. The study was carried out following the Declaration of Helsinki.

### Plasmid Constructs and Lentivirus Transduction

We used lentivirus vector pLKO.1-TRC-EGFP for carrying TRIM32 target shRNA and for non-silencing control. The sequence for TRIM32 shRNA was: GGUGGAAAGCUUUGGUGUU. Cells were transfected with the indicated plasmids by using calcium chloride.

### Real-Time Quantitative Polymerase Chain Reaction

RT-qPCR measured the mRNA expression of TRIM32. Total RNA was extracted from Ficoll-separated mononucleated bone marrow samples in AML patients and healthy donors using TRIzol reagents (Invitrogen, USA). Reverse transcription was performed at 25°C for 10 minutes, 42°C for 15 minutes, and 85°C for 5 minutes. qPCR was performed at 95°C for 10 min, followed by 40 cycles of amplification at 95°C for 15 s and 60°C for 60 s. The GAPDH mRNA was used as an internal control. The sequences of the oligonucleotides used in this study were as follows: TRIM32, forward, 5’-CTCGG-AAGTTCTTCACAGGCTC-3’ and reverse, 5’-CTCCAGTAGTGCTA-CATCTGCC-3’; GAPDH, forward, 5’-GAGAAGGCTGGGGCTCATT-T-3’ and reverse, 5’-ATGACGAACATGGG-GGCATC-3’.

### Western Blot

Cells were lysed on ice for 30 min with RIPA buffer (Beyotime) containing protease and phosphatase inhibitors (Beyotime). After centrifuging at 12,000g for 15 min at 4°C, the supernatant was re-suspended in buffer and heated at 100°C for 10 min. Protein quantification was performed by a BCA protein assay kit (Thermo). Equal amounts of proteins were electrophoresed in 10% sodium dodecyl sulfate (SDS) polyacrylamide gels and then transferred onto polyvinylidene difluoride (PVDF) membranes (Pierce, USA). After being blocked with 5% non-fat dried milk in Tris-buffered saline containing 0.1% Tween 20. The membranes were incubated at 4°C overnight with antibody TRIM32 (Abcam) and β-actin (Proteintech). After being incubated with appropriate secondary antibodies, proteins were visualized by a detection system of enhanced chemiluminescence (ECL).

### Cell Proliferation Assay

Proliferation assay was performed using a Cell Counting Kit-8 (CCK-8, Bimake). Appropriately 5 ×10^3^ cells/well were plated in a 96-well plate and cultured in complete medium. 10 μL of CCK8 reagent was added into each well and incubated for 2 hours at 37 °C with different time points of 0, 24, 48, and 72h. The absorbance was read at 450nm.

## Results

### Construction of Gene Modules by WGCNA

**Figure 1:**
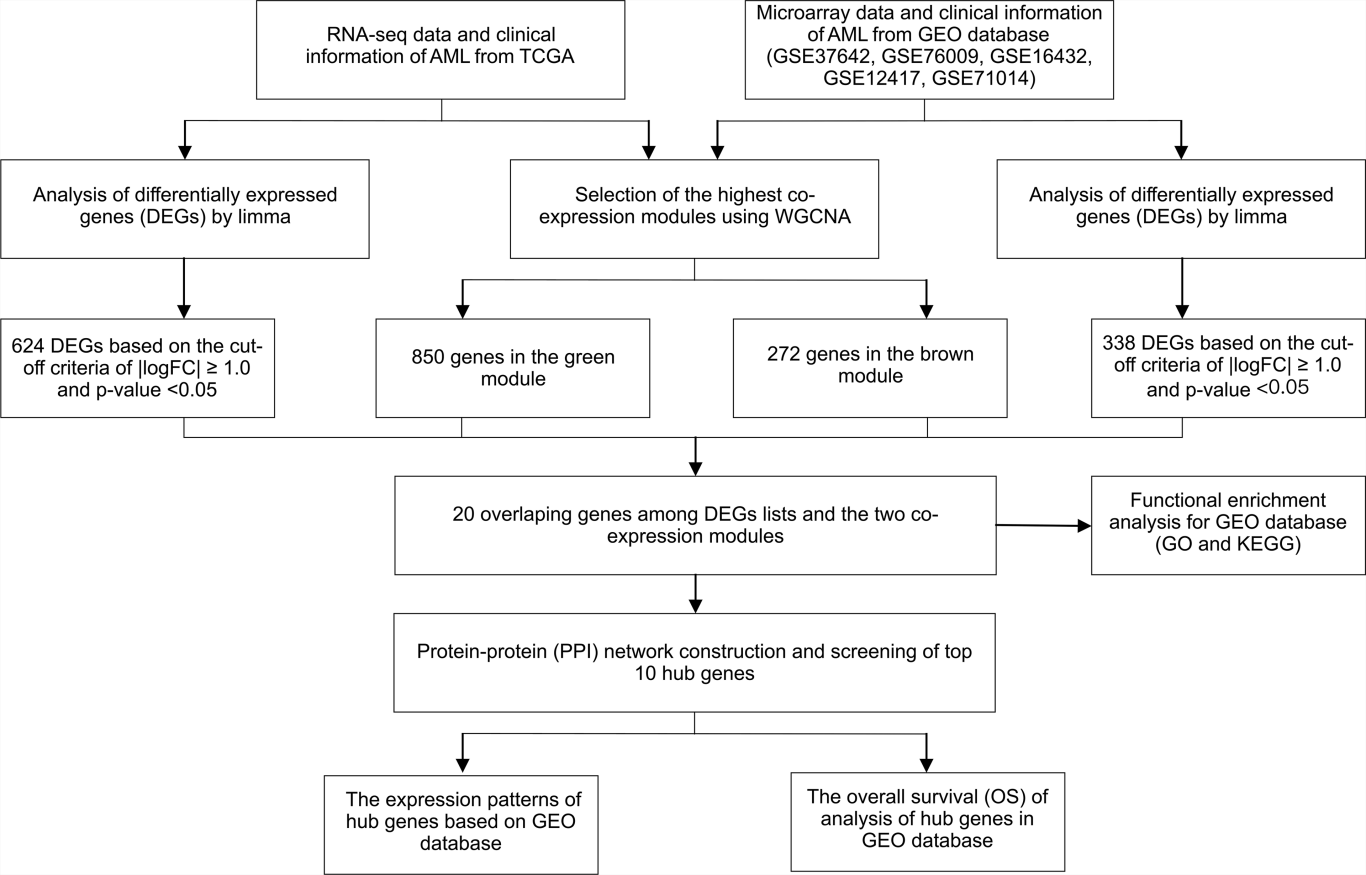
Study design and workflow of this study.

**Figure 2:**
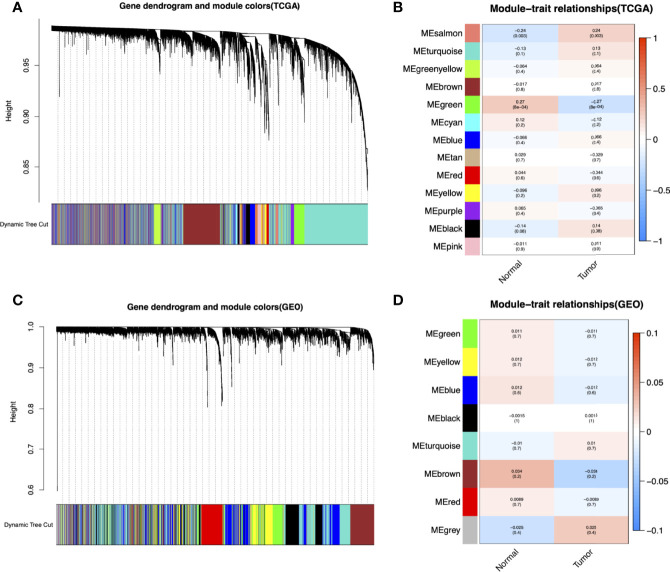
Identification of modules associated with the clinical information in the TCGA-LAML and GEO datasets (GSE37642, GSE76009, GSE16432, GSE12417, GSE71014). The Cluster dendrogram of co-expression network modules was ordered by a hierarchical clustering of genes based on the 1-TOM matrix in TCGA-LAML **(A)** and GEO datasets **(C)**. Each module was assigned different colors. Module-trait relationships in TCGA-LAML **(B)** and GEO datasets **(D)**. Each row corresponds to a color module and column corresponds to a clinical trait (cancer and normal). Each cell contains the corresponding correlation and P-value.

The flowchart of this study is shown in **Figure 1**. To identify key gene sets for AML, we performed a WGCNA from the TCGA database as well as the GSE37642, GSE76009, GSE16432, GSE12417, and GSE71014 datasets based on the *WGCNA* package (**Table S1**). Each color represents a module. Here, 13 unique modules in TCGA-LAML (**Figure 2A**) and 8 modules in the above five GEO datasets (**Figure 2C**) were identified. **Figures 2B, D** showed heat maps of module-trait relationships, indicating that the green modules in TCGA and the brown modules in the GEO dataset were highly distinguishable between tumors and normal individuals (green modules: r=0.27, *p*=8e-04, brown modules: r=0.034, *p*=0.2).

### Identification of Genes Between the DEGs and Co-Expression Modules

**Figure 3:**
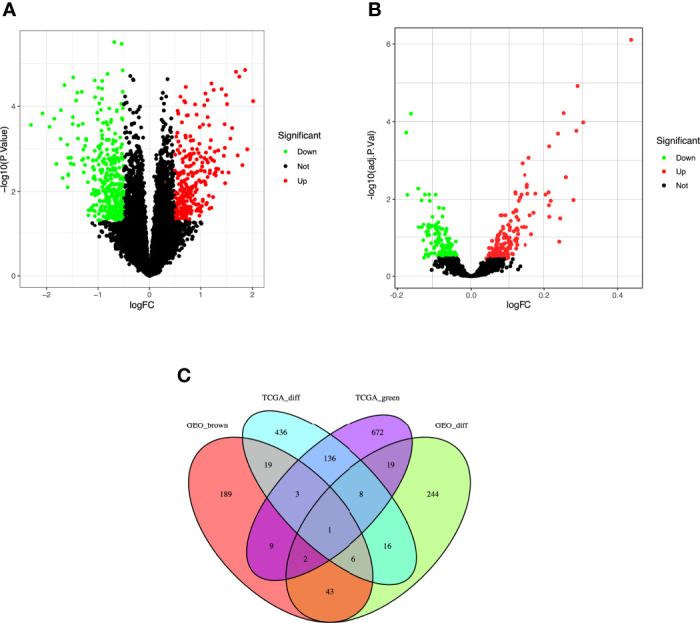
Identification of differentially expressed genes (DEGs) among the TCGA and GSE37642, GSE76009, GSE16432, GSE12417, GSE71014 datasets of AML with the cut-off criteria of |logFC| ≥ 1.0 and adj. P < 0.05. **(A)** Volcano plot of DEGs in the TCGA dataset. **(B)** Volcano plot of DEGs in the GSE37642, GSE76009, GSE16432, GSE12417, GSE71014 dataset. **(C)** The Venn diagram of genes among DEG lists and co-expression module. In total, 20 overlapping genes in the intersection of DEG lists and two co-expression modules.

A total of 624 DEGs in TCGA (**Figure 3A**) and 338 DEGs in the five GEO datasets (**Figure 3B**) were screened by the *limma* package with |logFC|≥1.0 and adj. p<0.05 as cut-off criteria. 1122 co-expressed genes were extracted from these significant modules based on WGCNA analysis (850 genes in the green module genes and 272 genes in the brown module). Finally, 20 overlapping genes were obtained at the intersection of the DEGs list and the two co-expression modules (**Figure 3C**).

### Function Enrichment Analysis for Genes in Co-Expression Module

**Figure 4:**
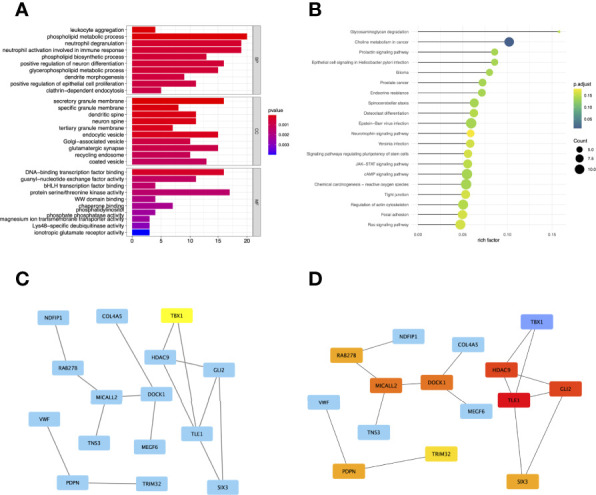
Gene Ontology (GO) **(A)** and Kyoto Encyclopedia of Genes and Genomes (KEGG) **(B)** enrichment analysis for the genes in the brown module. The color represents the adjusted p-values (BH), and the size of the spots represents the gene number. Visualization of the protein-protein interaction (PPI) network and the candidate hub genes. **(C)** PPI network of the genes between DEG lists and two co-expression modules. The blue nodes represent the genes. Edges indicate interaction associations between nodes. **(D)** Identification of the hub genes from the PPI network using maximal clique centrality (MCC) algorithm. Edges represent the protein-protein associations. The red nodes represent genes with a high MCC sores, while the yellow node represent genes with a low MCC sore.

To comprehensively understand the potential function of these genes in the co-expression modules, the GO (**Figure 4A**) and KEGG (**Figure 4B**) enrichment analyses for the genes in the brown module were performed using the R package “*ClusterProfiler*.” GO analysis divides genes into three categories: biological processes (BP), cellular components (CC), and molecular functions (MF). In BP, genes were enriched in phospholipid metabolic processes, neutrophil degranulation, and activation. In the CC category, many genes were involved in secretory granule membranes, endocytic vesicles, and glutamatergic synapses. In addition, protein serine/threonine kinase activity and DNA-binding transcription factor binding were mainly involved in MF. KEGG showed that genes were enriched in choline metabolism in cancer.

### Hub Genes Identified in the PPI Network

To investigate the correlation between DEGs and co-expression modules, we performed protein-protein interaction (PPI) analysis through the *STRING* database, and a PPI network containing 15 nodes and 15 edges was created in **Figure 4C**. In addition, the top 10 hub genes with MCC scores in the PPI network were calculated using Cytoscape’s CytoHubba plugin, which included TLE1, GLI2, HDAC9, MICALL2, DOCK1, PDPN, RAB27B, SIX3, TRIM32, and TBX1 (**Figure 4D**).

### Expression Level and Prognostic Value of the Hub Genes

**Figure 5:**
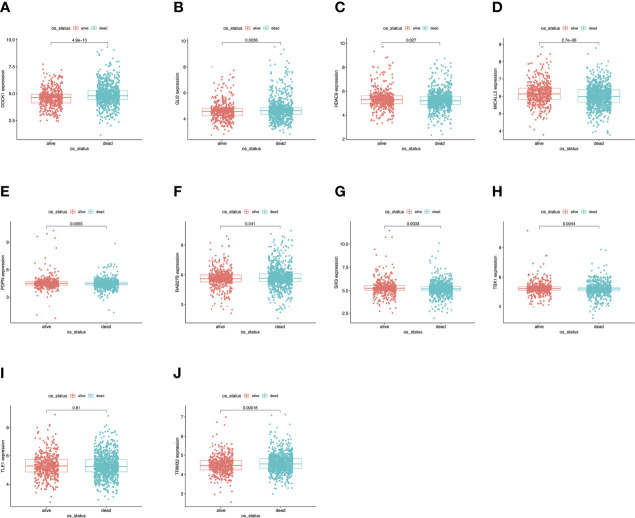
Validation of expression levels of the ten hub genes among AMLs from the GEO database. **(A)** Gene expression value DOCK1 among samples of GEO. **(B)** Gene expression value GLI2 among samples of GEO. **(C)** Gene expression value HDAC9 among samples of GEO. **(D)** Gene expression value MICALL2 among samples of GEO. **(E)** Gene expression value PDPN among samples of GEO. **(F)** Gene expression value RAB27B among samples of GEO. **(G)** Gene expression value SIX3 among samples of GEO. **(H)** Gene expression value TBX1 among samples of GEO. **(I)** Gene expression value TLE1 among samples of GEO. **(J)** Gene expression value TRIM32 among samples of GEO.

**Figure 6:**
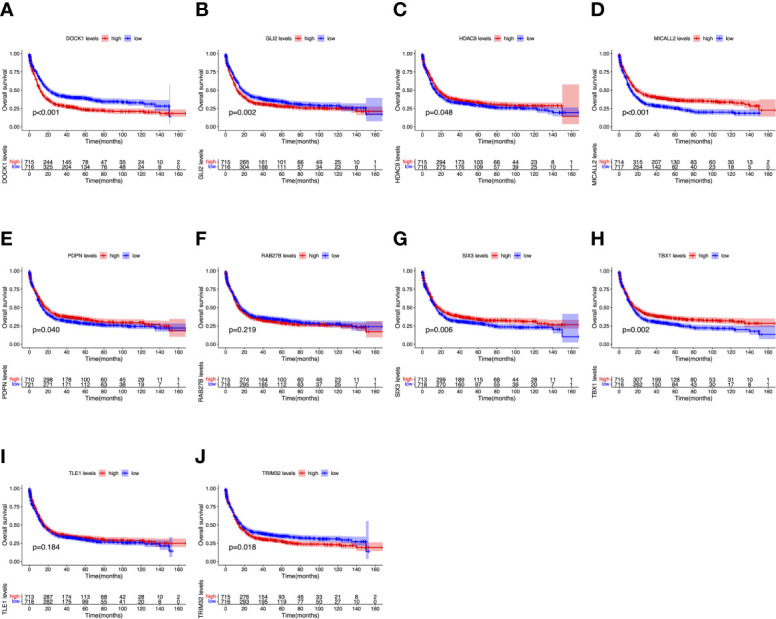
Overall survival (OS) analysis of ten hub genes in AML patients from the GEO database. **(A)** Survival analysis for DOCK1 in AML. **(B)** Survival analysis for GLI2 in AML. **(C)** Survival analysis for HDAC9 in AML. **(D)** Survival analysis for MICALL2 in AML. **(E)** Survival analysis for PDPN in AML. **(F)** Survival analysis for RAB27B in AML. **(G)** Survival analysis for SIX3 in AML. **(H)** Survival analysis for TBX1 in AML. **(I)** Survival analysis for TLE1 in AML. **(J)** Survival analysis for TRIM32 in AML. The patients were stratified into high-level group (red) and low-level group (blue) according to median expression of the gene. Log-rank P < 0.05 was a statistically significant difference.

To further determine the expression levels of hub genes in AML patients with different survival status (alive or dead), we mapped the expression in the GEO database. In addition to TLE1, four hub genes (DOCK1, GLI2, RAB27B, and TRIM32) were highly expressed in those who died, while the remaining five hub genes (HDAC, MICALL2, PDPN, SIX3, and TBX1) were expressed at lower levels (**Figure 5**). Then, to further explore the prognostic value of these ten hub genes, we performed a Kaplan-Meier survival analysis *via* the *survival* R package of the GEO dataset and was illustrated in **Figure 6**. The increased expression levels of DOCK1, GLI2, and TRIM32 were highly correlated with poor prognosis in AML (*p*<0.05).

### Validation of the Gene Expression and Prognosis of TRIM32

**Figure 7:**
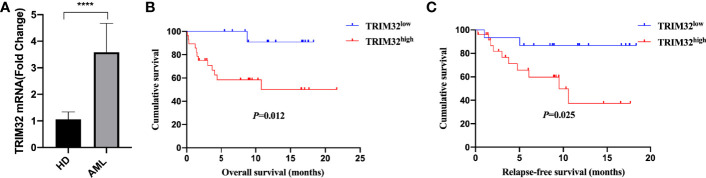
Validation of the gene expression and prognosis of TRIM32. **(A)** Expression of TRIM32 gene in 46 AML patients and 11 HD bone marrow samples by RT-qPCR. Overall survival **(B)** and relapse-free survival **(C)** between high and low expression of the gene TRIM32. **** means *P*<0.0001.

To date, the role of TRIM32 has not been reported in AML previously. Thus, we validated the role of TRIM32 with the clinical data of patients from our center. The mRNA levels of TRIM32 in 46 AML and 11 HD bone marrow samples were also detected by RT-qPCR. As shown in **Figure 7A**, TRIM32 expression was significantly upregulated in AML compared with that in HD (*p*<0.05). All patients were divided into two groups based on the optimal cut-off value of TRIM32 determined by the ROC curve (AUC: 0.682, sensitivity:91.7%, specificity: 43.3%) (**Figure S1**). The clinicopathological characteristics of these patients were summarized in **Table 1**. The expression of TRIM32 was correlated with biallelic CEBPA and PTPN11 mutation. Patients with lower expression of TRIM32 were more likely to achieve complete remission (CR) after the first induction therapy. Additionally, patients with elevated expression of TRIM32 were more frequently found in the adverse-risk group based on 2017ELN. The results of univariate and multivariate analyses of risk factors for OS were shown in **Table 2**. Older age, not achieving CR after first induction therapy, not receiving HSCT, gene mutation status (PTPN11^mt^, RUNX1^mt^ and IDH1^mt^) and high expression of TRIM32 were associated with poor OS in univariate analysis (**Figure 7B**). However, TRIM32 did not play an independent role in OS while RUNX1 mutation proved to be an independent risk factor for worse OS after multivariate analysis (HR:7.441, 95%CI: 1.595-34.712, *p*=0.011). Receiving HSCT was an independent protective factor (HR:0.104, 95%CI: 0.020-0.531, *p*=0.007). Similar results were observed for RFS shown in **Table 3**. Patients with overexpression of TRIM32 experienced a remarkably worse RFS in **Figure 7C** (*p*=0.025), although it was not an independent prognostic factor. Taken together, both the external database and clinical data in our center show that TRIM32 does have a certain influence on the prognosis of AML, whose role needs to be further studied.

**Table 1:** Relationship between the expression of TRIM32 and clinicopathological features.

Variables	Totalno.(%)	TRIM32^low^ (n = 15)	TRIM32^high^ (n = 31)	P value
Gender (n, %)				0.766
Male	29 (63.0)	9 (60.0)	20 (64.5)	
Female	17 (37.0)	6 (40.0)	11 (35.5)	
Age, years				0.519
Median (Range)	48 (17-78)	44 (17-65)	50 (22-78)	
WBC count, *10^9^/L				0.246
Median (Range)	19.6 (1.7-169.0)	14.75 (2.5-169)	22.21 (1.68-159.16)	
Hemoglobin, g/L				0.089
Median (Range)	72 (42-128)	90 (50-117)	70 (42-128)	
Platelet count, *10^9^/L				0.888
Median (Range)	50 (4-312)	46 (8-250)	51 (4-312)	
Blast in bone marrow (%)				0.605
<50	16 (34.8)	6 (40.0)	10 (32.3)	
≥50	30 (65.2)	9 (60.0)	21 (67.7)	
Karyotype				0.880
Favorable	4 (8.7)	1 (6.7)	3 (9.7)	
Intermediate	38 (82.6)	13 (86.7)	25 (80.6)	
Adverse	4 (8.7)	1 (6.7)	3 (9.7)	
Fusiongenes				0.419
Favorable	7 (15.2)	2 (13.3)	5 (16.1)	
Intermediate	37 (80.4)	13 (86.7)	24 (77.4)	
Adverse	2 (4.3)	0 (0.0)	2 (6.5)	
2017 ELN				0.062
Favorable	3 (6.5)	0 (0.0)	3 (9.7)	
Intermediate	27 (58.7)	12 (80.0)	15 (48.4)	
Adverse	16 (34.8)	3 (20.0)	13 (41.9)	
CR1				***0.005***
Yes	14 (30.4)	1 (6.7)	13 (41.9)	
No	25 (54.3)	13 (86.7)	12 (38.7)	
NA	7 (15.2)	1 (6.7)	6 (19.4)	
Biallelic CEBPA Mut^*^	10 (24.4)	6 (42.9)	4 (14.8)	***0.047***
DNMT3A Mut^*^	11 (26.8)	3 (21.4)	8 (29.6)	0.569
NPM1 Mut^*^	12 (29.3)	5 (35.7)	7 (25.9)	0.514
FLT3-ITD Mut^*^	15 (36.6)	5 (35.7)	10 (37.0)	0.934
FLT3-TKD Mut^*^	3 (7.3)	0 (0.0)	3 (11.1)	0.105
TET2 Mut^*^	7 (17.1)	3 (21.4)	4 (14.8)	0.598
EZH2 Mut^*^	3 (7.3)	2 (14.3)	1 (3.7)	0.232
NRAS Mut^*^	9 (22.0)	2 (14.3)	7 (25.9)	0.380
PTPN11 Mut^*^	5 (12.2)	0 (0.0)	5 (18.5)	***0.033***
RUNX1 Mut^*^	4 (9.8)	1 (7.1)	3 (11.1)	0.678
KIT Mut^*^	3 (7.3)	1 (7.1)	2 (7.4)	0.975
IDH1 Mut^*^	3 (7.3)	1 (7.1)	2 (7.4)	0.975
IDH2 Mut^*^	3 (7.3)	2 (14.3)	1 (3.7)	0.232

CR1, complete remission (CR) after first induction therapy. A P value of less than 0.05 is indicated in italics and bold. * Next generation sequencing data is missing from 5 patients. NA, notavailable.

**Table 2:** Univariate and multivariate analysis of risk factors for OS.

Variables	Univariate analysis			Multivariate analysis	
	No. of patients	1-year-OS (%)	P value	HR(95%CI)	P value
Gender (n, %)			0.960		
Male	25	73.3			
Female	17	62.7			
Age, years			***0.019***	——	0.089
<45	18	87.7			
≥45	24	52.3			
WBC count, *10^9^/L			0.224		
<100	38	65.6			
≥100	4	NA			
Hemoglobin, g/L			0.487		
<100	36	66.5			
≥100	6	80.0			
Platelet count, *10^9^/L			0.255		
<100	35	61.9			
≥100	7	66.7			
Blast in bone marrow (%)			0.611		
<50	15	66.1			
≥50	27	71.5			
Karyotype			0.420		
Fav/int	38	70.6			
Adv	4	50.0			
CR1^#^			***0.007***	——	0.458
Yes	14	86.0			
No	23	48.0			
Consolidation therapy			***0.001***	0.104(0.020-0.531)	***0.007***
HSCT	19	94.4			
Chemotherapy	23	26.6			
PTPN11^*^			0.064	——	0.400
Mut	5	40.0			
WT	33	74.5			
RUNX1^*^			***0.003***	7.441(1.595-34.712)	***0.011***
Mut	4	25.0			
WT	34	75.4			
IDH1^*^			0.076	——	0.093
Mut	3	33.3			
WT	35	73.8			
Expression of TRIM32			***0.012***	——	0.112
Low	14	90.9			
High	28	54.1			

A total of 42 patients were included in the OS analysis (4 patients were excluded for the lack of OS data). CR1, complete remission (CR) after first induction therapy. A P value of less than 0.05 is indicated in italics and bold. *Next generation sequencing data is missing from 4 patients with OS data. ^#^Response to induction chemotherapy was unknown in 5 patients with OS data. NA, not available.

**Table 3:** Univariate and multivariate analysis of risk factors for RFS.

Variables	Univariate analysis			Multivariate analysis	
	No. of patients	1-year-RFS (%)	P value	HR(95%CI)	P value
Gender (n, %)			0.307		
Male	15	73.0			
Female	25	47.4			
Age, years			***0.014***	——	0.148
<45	17	84.4			
≥45	23	48.2			
WBC count, *10^9^/L			0.195		
<100	36	58.5			
≥100	4	NA			
Hemoglobin, g/L			0.442		
<100	34	58.7			
≥100	6	83.3			
Platelet count, *10^9^/L			0.880		
<100	32	64.5			
≥100	8	60.0			
Blast in bone marrow (%)			0.793		
<50	13	65.3			
≥50	27	59.0			
Karyotype			0.974		
Fav/int	37	61.5			
Adv	3	66.7			
Consolidation therapy			***0.004***	0.175(0.047-0.658)	***0.010***
HSCT	19	42.4			
Chemotherapy	21	82.0			
PTPN11^*^			1.000		
Mut	4	66.7			
WT	33	61.7			
RUNX1^*^			0.451		
Mut	3	NA			
WT	34	61.0			
IDH1^*^			0.320		
Mut	3	NA			
WT	34	59.8			
Expression of TRIM32			***0.025***	——	0.128
Low	15	86.7			
High	25	44.5			

A total of 40 patients were included in the RFS analysis (6 patients were excluded for the lack of RFS data). A P value of less than 0.05 is indicated in italics and bold. * Next generation sequencing data is missing from 3 patients with RFS data. NA, not available.

### TRIM32 Is Highly Expressed in AML Cells and Promote AML Cell Proliferation *in Vitro*

**Figure 8:**
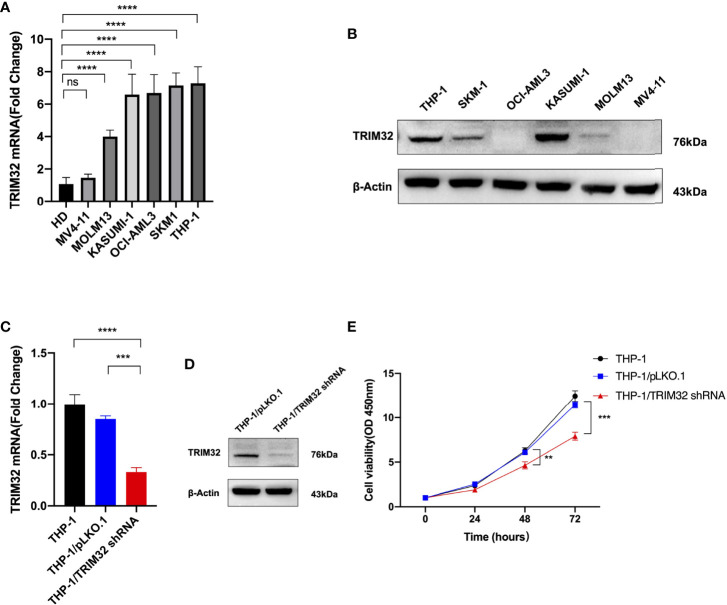
Expression level of TRIM32 was detected in six AML cell lines by RT-qPCR **(A)** and western blot **(B)**. Transfection efficiency of TRIM32 shRNA was validated in THP-1 using RT-qPCR **(C)** and western blot **(D)**. **(E)** Cell proliferation was determined by CCK-8 assay. ** means *P*<0.01, *** means *P*<0.001, **** means *P*<0.0001, ns, no significance.

We investigated the relative expression of TRIM32 mRNA levels in six AML cell lines (MV4-11, MOLM13, KASUMI-1, OCI-AML3, SKM1, THP-1) and bone marrow of HD (**Figure 8A**). TRIM32 was highly expressed in AML cells compared with HD. We also measured the protein level of TRIM32 in these cell lines (**Figure 8B**). To assess the potential role of TRIM32 in proliferation in AML cell lines, we chose THP-1 and KASUMI-1with higher expression of TRIM32 to stably knockdown TRIM32 using a lentiviral delivery system. The efficiency of the shRNA in THP-1 was verified by RT-qPCR (**Figure 8C**) and western blot (**Figure 8D**). Compared with the empty vector (pLKO.1), shRNA knockdown of TRIM32 significantly inhibited proliferation *in vitro* as indicated by CCK-8 assay (**Figure 8E**). Similar results were obtained in another AML cell line KASUMI-1 shown in **Figure S2**.

## Discussion

Acute myeloid leukemia (AML) is a highly malignant disease and remains the most common form in adults, accounting for about 32% of all adult leukemia cases (22). The underlying molecular mechanism of the initiation and progression of AML is still poorly understood (23). With the rapid development of high-throughput sequencing technologies, we can extract a large volume of genomic data from patient samples, contributing to a better understanding of the underlying mechanism and providing novel molecular targets for the treatment of AML.

In this study, a total of 2192 patients in TCGA and GEO datasets were analyzed. We obtained 20 overlapping genes in the intersection of DEGs lists and two co-expression modules (green and brown module) from TCGA and GEO datasets (GSE37642, GSE76009, GSE16432, GSE12417, and GSE71014) based on integrated bioinformatics analysis. Using GO and KEGG enrichment analysis by R package “*ClusterProfiler*”, genes were enriched in phospholipid and choline metabolic process. In addition, the 10 hub genes with the highest MCC scores in the PPI network were screened, including TLE1, GLI2, HDAC9, MICALL2, DOCK1, PDPN, RAB27B, SIX3, TRIM32, and TBX1. These hub genes were dysregulated in AML patients with different survival status (alive or dead). The higher levels of DOCK1, GLI2, and TRIM32 expression were closely associated with worse prognosis in AML. Ultimately, the expression levels and survival analysis of TRIM32 were validated. Knockdown of TRIM32 significantly inhibited proliferation of AML cell lines *in vitro*.

Nowadays, correlation networks are increasingly used in bioinformatics application (14). WGCNA has become a popular method for revealing the correlation patterns among genomic data and summarizing modules of highly correlated genes (24). WGCNA has been widely applied in various biological researches to identify candidate biomarkers or therapeutic targets, especially in cancers, e.g., breast cancer, hepatocellular carcinoma, and colorectal cancer (25–28). Several survival-specific lncRNAs and mRNAs of AML were identified based on WGCNA method with RNAseq data from TCGA (29). Chen et al. concluded that lncRNA-LOC646762 through the endocytosis signaling pathway might act as a survival biomarker for adult AML. Another study revealed that 15 hub genes were crucial for AML progression by comparing AML samples from TCGA with standard control samples from the Genotype-Tissue Expression (GTEx) database using WGCNA (30). Finally, overexpression of CEACAM5, one of the hub genes, was proved to be an unfavorable factor for prognosis.

In the present study, we focused on the hub gene TRIM32 since both DOCK1 and GLI2 had been discussed as adverse prognostic markers of AML in previous articles (31–34). To the best of our knowledge, it is the first time to report and validate the role of TRIM32 in AML. Tripartite motif protein (TRIM) is a highly conserved and rapidly evolving family, which plays a vital role in cell growth, apoptosis and immunity. TRIM32 is a member of the TRIM family, first discovered in 1995 as a ubiquitin ligase enzyme (E3) (4). Actin, c-Myc, and ABI2 (Ab1 interactor 2) have been proved to be substrates for TRIM32 (35–37). TRIM32 is implicated in diverse diseases, especially in malignancies (9, 11, 38, 39). According to previous studies, aberrant overexpression of TRIM32 was depicted in human skin cancer cells (40). Moreover, TRIM32 was a significant predictor of the prognosis of hepatocellular carcinoma (HCC), and the overexpression of TRIM32 may induce HCC patients’ resistance to oxaliplatin (11). Liu et al. identified TRIM32 as a novel tumor suppressor p53 target gene and negatively regulated p53-mediated stress responses (41). TRIM32 overexpression promoted cell oncogenic transformation and tumorigenesis in mice in a largely p53-dependent manner. Another study found that TRIM32 can enhance retinoic acid receptor α (RARα)-mediated transcriptional activity and stabilize RARα in human promyelogenous leukemia cell line HL60 (42). Based on integrated bioinformatics analysis, our study is the first to reveal that TRIM32 is highly expressed in human AML, correlating with a poor prognosis. Furthermore, we validated the finding in human AML cell lines and patient samples in our center. Previous studies demonstrated that overexpression of TRIM32 could promote proliferation of lung cancer cells by activating JAK2/STAT3 signaling pathway (38). Both abnormal activations of JAK kinase and STAT are involved in multiple hematological malignant, especially leukemia (43). Recently, a variety of studies have proved that TRIM32 is closely related to glucose metabolism both in normal and tumor tissues by interaction with two enzymes involved in glycolysis (44). Elevated expression of TRIM32 contributes to a high rate of glycolysis with increased lactate production, which is like the Warburg effect in tumors. Metabolic reprogramming is also common in AML. However, the mechanism of oncogene TRIM32 in AML needs to be further verified by a series of fundamental experiments in the future.

In conclusion, we discovered ten hub genes enriched in human AML through integrated bioinformatics analysis. Based on WGCNA and differential gene expression analysis, TRIM32 was identified to be closely related to human AML, which could be used as a potential therapeutic target and prognostic biomarker for AML in the future.

## Data Availability Statement

The datasets presented in this study can be found in online repositories. The names of the repository/repositories and accession number(s) can be found in the article/**Supplementary Material**.

## Ethics Statement

The studies involving human participants were reviewed and approved by Medical Ethics Committee of the First Affiliated Hospital of Soochow University. The patients/participants provided their written informed consent to participate in this study.

## Author Contributions

XX and JQ designed the study, analyzed the data, and wrote the manuscript draft. JY contributed to searching the database. TP, HH and MY contributed to data analysis. YH participated in the whole process of this study, supervised and revised the manuscript. All authors contributed to the article and approved the submitted version.

## Funding

This work was supported by National Natural Science Foundation of China (81873432 and 82070143), grants from the Jiangsu Province of China (BE2021645), and the Priority Academic Program Development of Jiangsu Higher Education Institutions (PAPD).

## Conflict of Interest

The authors declare that the research was conducted in the absence of any commercial or financial relationships that could be construed as a potential conflict of interest.

## Publisher’s Note

All claims expressed in this article are solely those of the authors and do not necessarily represent those of their affiliated organizations, or those of the publisher, the editors and the reviewers. Any product that may be evaluated in this article, or claim that may be made by its manufacturer, is not guaranteed or endorsed by the publisher.
