# Supplementary material for: Up-Regulation of TRIM32 Associated With the Poor Prognosis of Acute Myeloid Leukemia by Integrated Bioinformatics Analysis With External Validation
Source: Front Oncol. 2022 Jun 8;12:848395. doi: 10.3389/fonc.2022.848395 (PMC9213666; doi:10.3389/fonc.2022.848395)
Supplement: Supplementary file 1 [file DataSheet_1.docx]

| **Supplemental Table 1**. Data collected from GEO and TCGA databases | | | | | | |
| --- | --- | --- | --- | --- | --- | --- |
|  | Author | Year | Series.No | Sample Size | Contains OS data | Application |
| TCGA | TCGA_GDC | 2021 | LAML | 151 | 151 | - |
| GEO | Herold T et.al | 2013 | GSE37642 | 562 | 422 | Haematologica 2018 Mar;103(3):456-465.  Blood 2014 Aug 21;124(8):1304-11.  J Clin Oncol 2013 Mar 20;31(9):1172-81. Sci Rep 2015 Dec 17;5:18411. |
|  | Ng SW et.al | 2016 | GSE76009 | 534 | 307 | Nature 2016 Dec 15;540(7633):433-437. |
|  | Bullinger L et.al | 2010 | GSE16432 | 436 | 436 | Nat Med 2010 Aug;16(8):903-8. Oncotarget 2017 Nov 10;8(56):95038-95053. |
|  | Metzeler KH et.al | 2008 | GSE12417 | 405 | 242 | Blood 2008 Nov 15;112(10):4193-201.  Ann Hematol 2021 Feb;100(2):487-498. |
|  | Chou W et.al | 2017 | GSE71014 | 104 | 104 | Ann Hematol 2021 Feb;100(2):487-498.  Oncotarget 2017 Sep 22;8(42):72250-72259.  Oncotarget 2015 Nov 17;6(36):39098-110. |


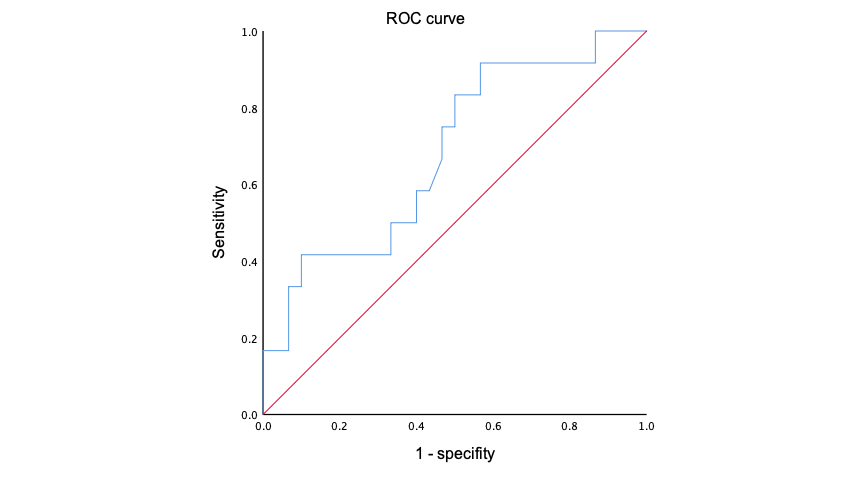


**Supplemental Figure 1.** Receiver operating characteristics (ROC) curve of TRIM32 based on the patient survival status.


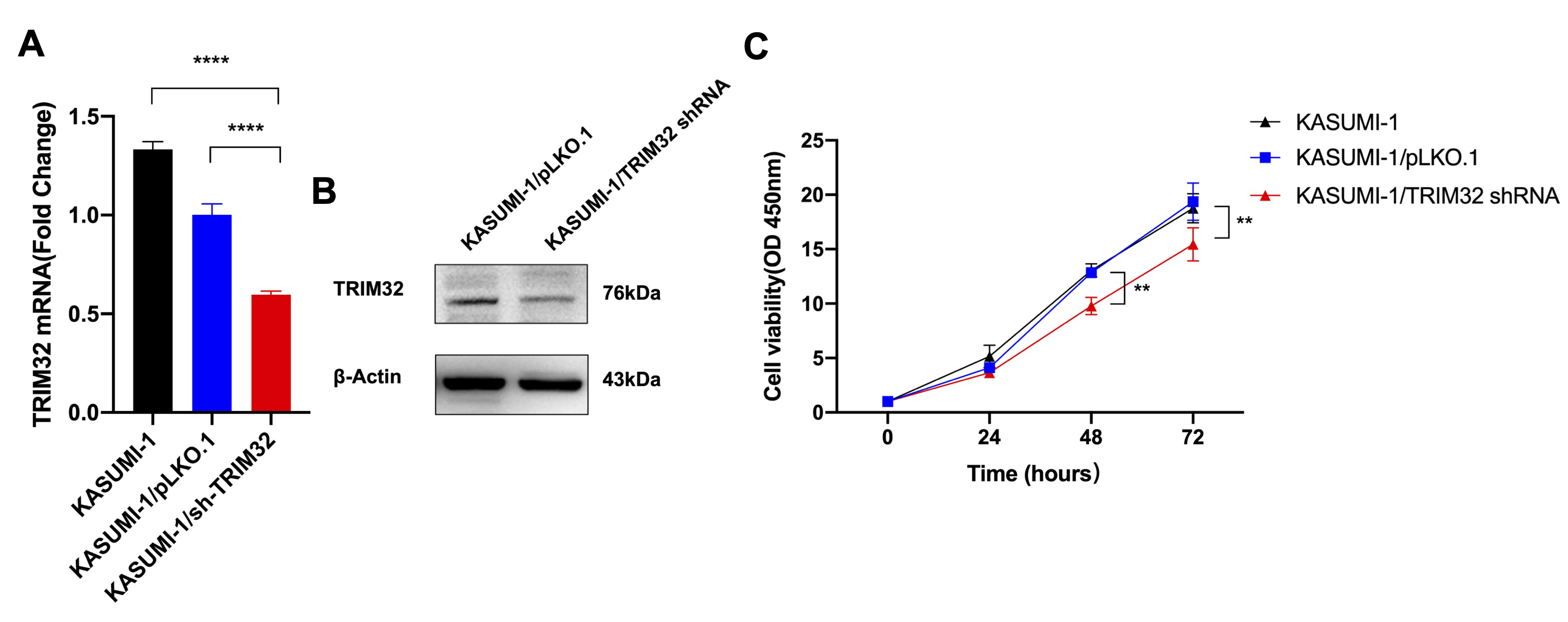


**Supplemental Figure 2.** Transfection efficiency of TRIM32 shRNA was validated in KASUMI-1 using RT-qPCR (A) and western blot (B). (C) Cell proliferation was determined by CCK-8 assay.
